# *QuickStats*: Percentage of Emergency Department Visits* with Medicaid as the Primary Expected Source of Payment Among Persons Aged <65 Years, by Race and Ethnicity^†^ — National Hospital Ambulatory Medical Care Survey, United States, 2011–2021

**DOI:** 10.15585/mmwr.mm7231a6

**Published:** 2023-08-04

**Figure:**
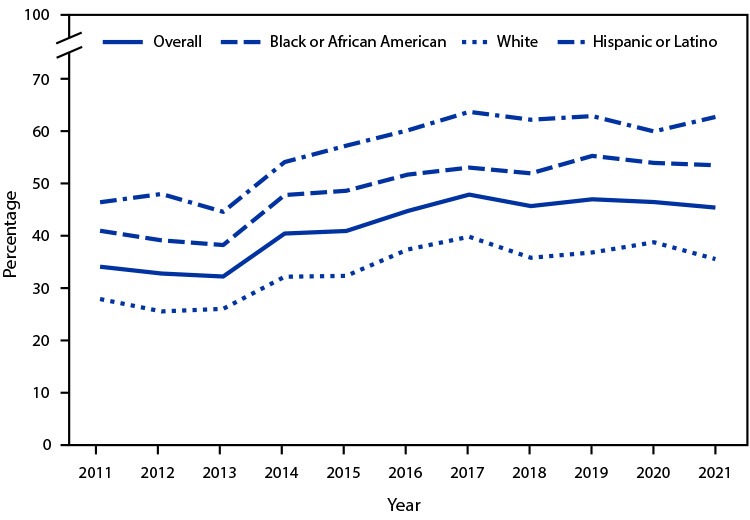
During 2011–2021, the percentage of ED visits among persons aged <65 years with Medicaid as the primary expected source of payment increased from 34.0% to 45.3%. This pattern was consistent irrespective of race and Hispanic or Latino (Hispanic) origin. ED visits among Hispanic persons increased the most, from 46.3% in 2011 to 62.7% in 2021. The percentage of ED visits among persons with Medicaid as their primary expected source of payment increased from 40.9% in 2011 to 53.4% in 2021 among Black or African American (Black) persons, and from 27.8% to 35.5% among White persons. During the study period, the percentages of ED visits among Black and Hispanic persons with Medicaid as the primary expected source of payment were higher than the percentages of visits by White persons.

**Source:** National Center for Health Statistics, National Hospital Ambulatory Medical Care Survey, 2011–2021. https://www.cdc.gov/nchs/ahcd/ index.htm

